# Oral myiasis

**DOI:** 10.11604/pamj.2022.41.335.32098

**Published:** 2022-04-26

**Authors:** Nalini Aswath

**Affiliations:** 1 Department of Oral Medicine and Radiology, Sree Balaji Dental College and Hospital, Bharath Institute of Higher Education and Research, Chennai-600100, Chennai, India

**Keywords:** Dipteran larvae, oral myiasis, teeth

## Image in medicine

Myiasis is a term derived from the Greek word “myia,” meaning invasion of vital tissue of humans or other mammals by fly larvae. Flies causing myiasis belong to the order Diptera. Oral myiasis is a rare pathology that occurs consequent to poor oral hygiene secondary to poor maintenance as seen in elderly, chronic hospitalized, psychiatric patients, contaminated wound, deleterious oral habits etc. It is associated with severe halitosis. The larvae or maggots invade the body tissues and cause destruction. Treatment includes flushing the affected area with turpentine, manual removal of maggots with tweezers, followed by a saline wash and irrigation of the affected area with betadine. An elderly male patient reported to the hospital with complaints of worms in the region of the upper anterior teeth. The patient gave a history of accidental fall on a mud road after intake of alcohol. On intra oral examination, the patient´s oral hygiene was very bad and a raw unhealed ulcer infested with maggots was noticed in the palatal aspect of the maxillary anterior teeth. The teeth were periodontally weak and grossly mobile. The mobile teeth were extracted. The larvae were flushed with turpentine, followed by wound cleansing with saline and irrigation with betadine.

**Figure 1:**
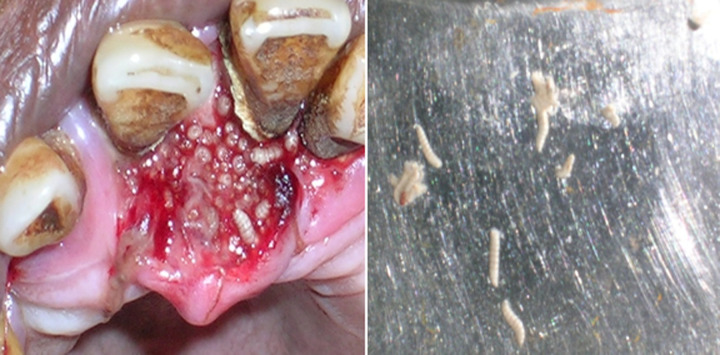
raw unhealed ulcer infested with maggots in the palatal aspect of the maxillary anterior teeth

